# Vaccination in the Light of the Royal British Commission

**Published:** 1898-05

**Authors:** M. R. Leverson

**Affiliations:** Fort Hamilton, L. I., N. Y.


### VACCINATION IN THE LIGHT OF THE ROYAL BRITISH COMMISSION.

M. R. Leverson, M. D., Fort Hamilton, L. I., N. Y.

(Continued from April No., p. 147.)

Q. 4,772. Dr. Cory is not familiar with the pamphlet, "Facts Concerning Vaccination for the Heads of Families," which is stated to have been revised by the Local Government Board and issued with their sanction.

Q. 4,774. (Dr. Bristowe). "Can you give us any information at all with regard to the views of those who have investigated the cow-pox; on what grounds were you induced to use the lymph from it?" "Dr. Klein brought up some of the cow-pox lymph and we vaccinated calves with it, producing quite characteristic-looking vesicles; and then I brought some of the lymph from those calves down and used it on some of the children at St. Thomas."*

Q. 4,775. Dr. Cory thinks that vaccine virus does not deteriorate by going from human being to human being if properly managed.

Qs. 4,776-80. In reply to Sir Edwin Galsworthy, Dr. Cory describes the mode in which he instructs and examines students and candidates for certificates as vaccinators.

Q. 4,781. The change to calf lymph from humanized lymph, which was merely a renewal of our stock, was made at the Surrey Chapel Station more as a matter of convenience than anything else.

Q. 4,792-4. Dr. Cory thinks there is less inflammation when human lymph than when calf lymph is used; but he has not worked the question out carefully.

---

* This is a remarkable statement to be given as an answer to Dr. Bristowe's question. Let it be remembered that Dr. Cory is the English government's "Instructor in Vaccination." Further, it nowhere appears to what cow-pox lymph Dr. Klein here refers, but it seems probable that it is the stuff spoken of in Qs. 4,630–4,637 and Qs. 4,783–4,789.

Q. 4,797. Repeats that it is a matter of indifference whether re-vaccination be performed with human lymph or calf lymph; the result is the same.

Q. 4,798 (To Mr. Hutchinson). Dr. Cory has no reason for the preference of calf lymph to human lymph.

Q. 4,799. "Is one followed by irritation more frequently than the other?" "I think not; I do not think that there is any real difference."

Q. 4,802 (Prof. Michael Foster). "When you said in answer to Q. 4,338 that it was a matter of comparative indifference which lymph was used, were you taking into consideration the probability of inflammation and other untoward consequences, or was your attention in answering the question directed only to the efficiency of the vesicle?" "I was speaking then with regard to the vesicle, I think; at least that was my impression at the time."

Q. 4,804. "Such differences as there may be between calf and human lymph with regard to the development of untoward results are so small that you still regard them as a matter of indifference?" "I think so."

Q. 4,805 (Dr. Collins). "Then would your answer to Q. 4,491 be correct where you say, 'My impression is that you get more sore arms after using calf lymph than after using humanized lymph?'" *"I am afraid that I have contradicted myself there, as in many other places."**

---

* The ingenuousness of this answer, together with the sincerity of his belief, which he demonstrated by syphilizing himself by vaccination so thoroughly that it was feared his arm would have to be amputated in his vain effort to prove that syphilis could not be invaccinated, led me to regard Dr. Cory in a more favorable light than I did Drs. Simon, Thorne and other official witnesses, as may be seen on pp. 497–8 of Vol. XVI. of THE HOMŒOPATHIC PHYSICIAN, in which the present abstract of the testimony taken by the Royal British Commission was commenced.

In view of the remarkable record of Dr. Cory's evidence now given, of the recklessness it displays of infantile suffering and life, and of his shifty and evasive replies, the reader must decide whether the opinion before expressed was not far too lenient, and whether a closer study of Dr. Cory's testimony must not lead to a far less charitable judgment.

Q. 4,808 (Mr. Whitbread). "Quite accepting your explanation of the way in which the plural 'hospitals' got into the card, do you think it prudent to issue that card with a statement on it confined to the one hospital, when, as a matter of fact, it has been proved that re-vaccination does not give such absolute immunity?" "I do not know that it would really make very much difference if you left the 's' in. There would be very few, if any, cases where a nurse who has had small-pox has been previously successfully re-vaccinated."

Q. 4,809 (Dr. Collins). "Are you aware that it is stated in a pamphlet, which has been issued with the sanction of the Local Government Board, that soldiers who have been re-vaccinated can live in cities intensely affected by small-pox without themselves suffering to any appreciable degree from that disease?" "Yes, I know that statement."

Q. 4,810. "Evidence has come before the Commission that the reason for the excess of small-pox in the army in India, which is re-vaccinated, is their greater exposure to contagion?" "*I know nothing of the army in India.*"

As above mentioned, Dr. Cory appeared again before the Commission on the 7th of May, 1890. What he then said concerning his "contradictions" regarding the Wiltshire or Alderley (Gloucestershire) cow-pox and lymph was abstracted so as to follow immediately his testimony of December 4, 1889. We will now proceed to abstract the rest of his evidence of the later date.

Q. 8,912 (Dr. Collins). "In answer to Q 4,660, 'Would you approve of that statement that the perfect character of the vesicle is no guarantee that it will not furnish both vaccine and syphilitic virus,' you replied, 'Yes, I should approve of it.' That answer *has since been altered* to 'I do not disapprove of it.' Would you wish to qualify that answer in any way so that we may understand your view?" "Not further than that."

Qs. 8,913-14. Quoting from Dr. Ballard's essay on vaccination, p. 345, "the perfect character of a vaccine vesicle is no guarantee that it will not furnish both vaccine and syphilitic virus," Dr. Cory says "that statement is correct, and I do not disapprove of that opinion."

Q. 8,915. "That to my mind expresses a limited agreement. I thought you might perhaps tells us what the limitation was that you wished to make?" "No, I have nothing to add to that statement."

Q. 8,916. "Do you or do you not fully approve of that statement that 'The perfect character of the vesicle is no guarantee that it will not furnish both vaccine and syphilitic virus?' " "I cannot say more than that I do not disapprove of it."

Q. 8,917. "Do you approve of the statement that 'It is possible for syphilis to be communicated in vaccination from the vaccine vesicle upon a syphilitic person, notwithstanding the operation being performed with the utmost care to prevent the admixture of blood?" "But the patient must be suffering from the early symptoms of syphilis for that effect to be produced."

Q. 8,918. He says that almost all children with syphilis have not received the infection directly, but they have inherited it. The first eruption of their syphilis would be a dangerous time for taking vaccine lymph from them.

Q. 8,919. He believes it is possible to convey syphilis from an infant subject to hereditary syphilis, though there be no blood in the lymph. When the syphilis is latent it is not infectious in vaccination,* and so far as he knows not after a certain lapse of time.

Q. 8,920. Dr. Cory does not know of any cases on record

---

* Neither biology, physiology nor pathology affords the least warrant for this statement—as rash a one as ever promulgated by any physician. The history and pathology of vaccination afford many facts tending to the contrary conclusion, and which would be far more consonant with the teachings of the sciences above mentioned than Dr. Cory's bold assertion. M. R. L.

showing syphilis to have been invaccinated from children subjects of hereditary disease without the vaccinifer at the time manifesting definite evidence of syphillis.

Q. 8,921. He says he is familiar with Mr. Hutchinson's well-known series.

Q. 8,922. "Were not there amongst his series cases in which individuals were infected with syphilis by vaccination, the vaccinifer not at the time showing definite evidence of syphilis?" "I was hardly prepared to be questioned upon these points."

Qs. 8,923-4. Has read the description by Prof. Crookshank of the early history of the outbreak at Laforet from which the Lamb's Conduit Street stock is derived—there was more than one outbreak at the time, or shortly afterwards.

Q. 8,925. "Have you formed any opinion as to whether those outbreaks were of true or spurious cow-pox?" "Certainly; the lymph we got from the first outbreak was vaccinia."

Q. 8,926. "Are you aware that Dr. Layet, speaking of these outbreaks, says that the dissimilarity of the eruption of the different cows is certified by Dr. Landeau and Dr. Pujos, who had been there and seen the eruptions. Hence the following conclusion: 'Are there several eruptive maladies of the bovine species which are capable of furnishing true vaccine?' And, again: "There is anything but agreement about what is called spontaneous cow-pox?" "I, of course, can only speak of the lymph which we got over to England which I saw, and that lymph was undoubtedly vaccine."

Q. 8,927. "Judged by what?" "From its giving protection from small-pox."

Qs. 8,928-9. As employed upon the nurses at the Highgate Hospital.

Qs. 8,930-1. He is not familiar with Bousquet's work on cow-pox, but admits he is an authority.

Q. 8,932. "Do you know that he says that neither in general nor in the local symptoms, which vary immensely, is there anything definite by which to discriminate the true from the spurious cow-pox?" "Of course, I cannot be answerable for other people's opinions. I form my own from my experience."*

Sir William Savory, Prof. Michael Foster, and the Chairman then put questions to Dr. Cory to lead him to explain away his contradictory and shifty testimony; but the cloud thus attempted to be thrown over his confusion is dissipated by a few searching questions put by Mr. Bradlaugh. It is not therefore worth while to cumber the pages of THE HOMŒOPATHIC PHYSICIAN with them, unless it were for the purpose of making clear the prejudiced and partial character of the tribunal by whom the investigation was being conducted. It will be sufficient, for this purpose to refer the student of the subject, *or of human nature*, to Qs. 8,934-8,948.

Q. 8,949 (To Dr. Bristowe). It was from a passage Dr. Cory saw in one of the reports of the Cattle Plague Commission that Dr. Cory formed his opinion that inoculation of cattle plague upon a human being might produce local results like those of cow-pox.

Qs. 8,960-2 (To Mr. Bradlaugh). Doctors might disagree as to whether a child born of parents who had had syphilis was a proper vaccinifer; but if it did not present any symptoms of syphilis at the time he would regard the child as quite safe.

Qs. 8,963-4. It is quite possible that a child might not present any symptoms of syphilis at the time the lymph was

* In the twelfth annual report of the Local Government Board, 1883, page 49, we find it stated: "It is conclusively proved by Dr. Cory's experiments that it is possible for syphilis to be communicated in vaccination from a vaccine vesicle on a syphilitic person notwithstanding that the operation be performed with the utmost care to avoid the admixture with blood."

taken from him, and yet within six months might exhibit serious symptoms of syphilis and die from syphilis.

Q. 8,965. He thinks that a child who was so affected by hereditary syphilis as to die within the first year from syphilis could be safely used as a vaccinifer, because it was apparently healthy at the time.

Qs. 8,969-70. At Q. 5,089 of the testimony taken before the Select Committee in 1871 Mr. Hutchinson was asked, "Have you any security whatever that the child from whom you take the lymph may not be suffering from syphilis?" to which he replied: "I believe that there are cases of latent syphilis which cannot be detected by any medical man unless he examines into the history of the child as well as its appearance." Dr. Cory agrees with that.

Q. 8,972. "Would you kindly read the next question and answer?" (Q.) "Then, notwithstanding any amount of skill or care on the part of the practitioner, he may, if he touches blood, communicate syphilis, may he not?" (A.) "I am obliged to say that I believe he may, but I believe it would be exceedingly infrequent."

Q. 8,973. Dr. Cory does not agree in that, because (Q. 8,974) he believes that when the symptoms of syphilis are latent it is not inoculable in vaccination.*

Q. 8,975 (Dr. Collins). "Would you say in reference to plate 17 in Prof. Crookshank's book, *The History and Pathology of Vaccination*, that that which is described as 'casual cow-pox' is incorrectly named, as far as you can judge from the appearances there represented?" "I should not like to say."

Q. 8,977. "Are the appearances there represented similar to those which result from the inoculation of true cow-pox

---

* W. Hutchinson, in his *Archives of Surgery*, for October, 1890, says: "It is absurd to assert that inherited syphilis is always to be detected, and it is cruel injustice to imply that all accidents have been the result of carelessness."

on a milker's hands?" "I have never seen true cow-pox on a milker's hands from the casual disease of the cow."

We have at last done with the testimony of Dr. Cory. I regret to have had to give such copious extracts from it, but had I done less, or had I attempted to present a picture of that testimony otherwise than in his own words, I should either have failed altogether in giving the slightest idea of its character, or else would have invited from the enraged adherents of vaccination flat denials and accusations of wilful misrepresentation.

The next witness was Dr. Charles Creighton.

